# Crystal structure and Hirshfeld surface analysis of *N*,*N*′-[ethane-1,2-diylbis(­oxy)]bis­(4-methyl­benzene­sulfonamide)

**DOI:** 10.1107/S2056989018017437

**Published:** 2019-01-01

**Authors:** Seher Meral, Sevgi Kansiz, Necmi Dege, Aysen Alaman Agar, Galyna G. Tsapyuk

**Affiliations:** a Ondokuz Mayıs University, Faculty of Arts and Sciences, Department of Chemistry, 55139, Samsun, Turkey; b Ondokuz Mayıs University, Faculty of Arts and Sciences, Department of Physics, 55139, Kurupelit, Samsun, Turkey; c Taras Shevchenko National University of Kyiv, Department of Chemistry, 64, Vladimirska Str., Kiev 01601, Ukraine

**Keywords:** crystal structure, amine, benzene­sulfonamide, Hirshfeld surface, hydrogen bonding

## Abstract

In the mol­ecule of the title compound, C_16_H_20_N_2_O_6_S_2_, the mid-point of the C—C bond of the central ethane moiety is located on a twofold rotation axis. In the crystal, mol­ecules are linked by N—H⋯O hydrogen bonds into supra­molecular chains propagating along the [101] direction. Hirshfeld surface analysis and two-dimensional fingerprint plots indicate that the most important contributions to the crystal packing are from H⋯H (43.1%), O⋯H/H⋯O (40.9%), C⋯H/H⋯C (8.8%) and C⋯C (5.5%) inter­actions.

## Abstract 2

In the crystal, the mol­ecules are linked by N—H⋯O hydrogen bonds into supra­molecular chains propagating along the [101] direction.

## Chemical context   

Sulfonamides are synthetic mol­ecules which include the SO_2_–NH group and are called sulfa drugs. These effective drug mol­ecules have an important role in the medical field, including as promising chemotherapeutic agents, and have been used in the treatment of many bacterial infections due to their physical, chemical and biological properties (Mahmood *et al.*, 2016 ▸; Ghorab *et al.*, 2018 ▸). Recently, sulfonamides have also been used in the organic synthesis reactions for the synthesis of linear or cyclic oligomers and the introduction of nucleophilic heteroatom functionality to the synthesized mol­ecule (Ni *et al.*, 2015 ▸). *N*,*N*′-di­tosyl­alkane di­amine is a disulfonamide synthesized by the tosyl­ation of di­amine, and this synthetic mol­ecule has anti­bacterial properties (Alyar *et al.*, 2011 ▸) and has also been used in many organic synthesis reactions (Rong *et al.*, 1998 ▸). In this study, the synthesis, crystal structure and Hirshfeld surface analysis are reported for the new potential sulfa drug, *N*,*N*′-[ethane-1,2-diylbis(­oxy)]bis­(4-methyl­benzene­sulf­on­a­mide).
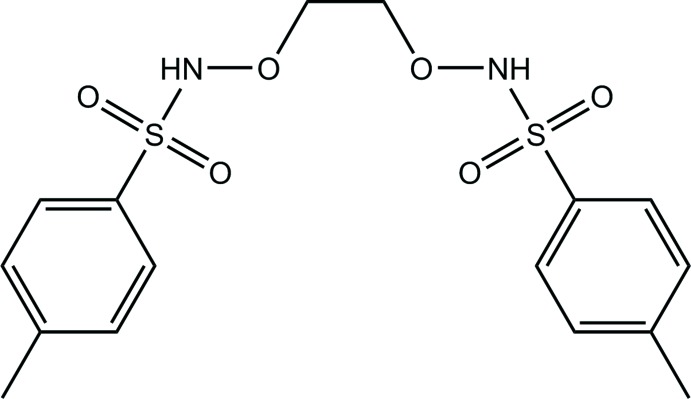


## Structural commentary   

**Figure 1:**
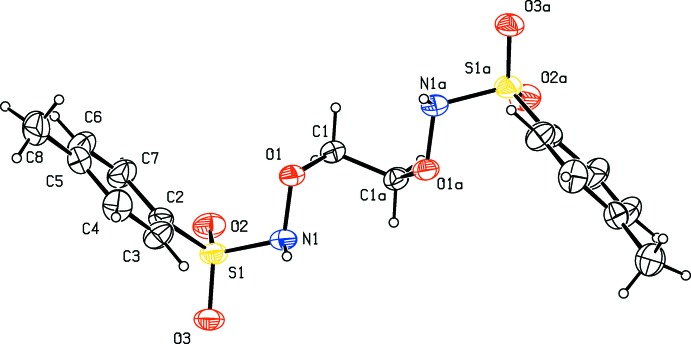
The mol­ecular structure of the title compound, showing the atom labelling. Displacement ellipsoids are drawn at the 20% probability level. Symmetry code: (a) −*x*, *y*, −*z* − 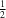.

The mol­ecular structure of the title compound is illustrated in Fig. 1 ▸. The mol­ecular point group symmetry is *C_2v_* (*mm*2) (H atoms excluded), with the twofold rotation axis bisecting the central C1—C1^i^ bond. The mol­ecule is Z-shaped with the N1—S1—C2—C3 torsion angle being −60.6 (3)°. The C1—O1 bond length of 1.429 (3) Å and the O1—N1 bond length of 1.426 (2) Å are close to the values reported for similar compounds (see the *Database survey*). The S1—O2 and S1—O3 distances are 1.4376 (17) and 1.4168 (19) Å, respectively while the S1—N1 and S1—C2 distances are 1.647 (3) Å and 1.747 (3) Å, respectively.

## Supra­molecular features   

**Figure 2:**
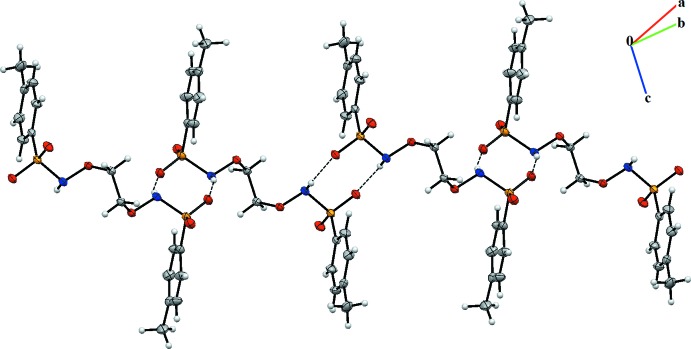
A view of the chain structure formed by N—H⋯O hydrogen bonding.

**Figure 3:**
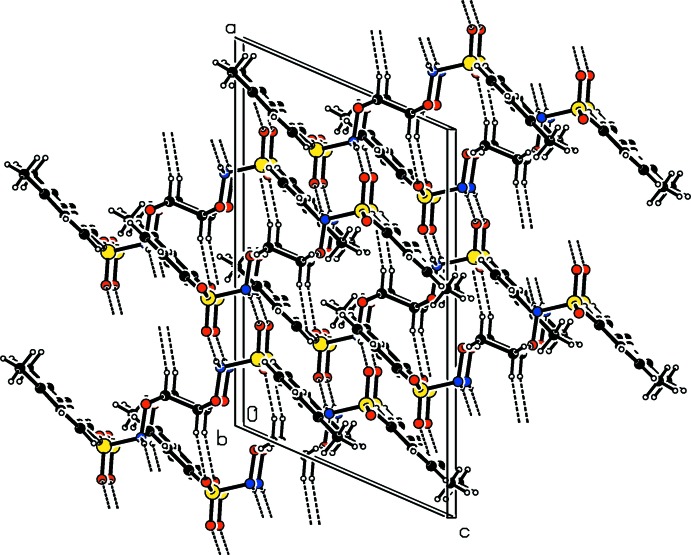
A view along the *b*-axis of the crystal packing of the title compound. The hydrogen bonds (Table 1 ▸) are shown as dashed lines.

The crystal packing of the title compound features inter­molecular N—H⋯O hydrogen bonds (Table 1 ▸ and Fig. 2 ▸), which connect the mol­ecules into supra­molecular chains propagating along the [101] direction. The chains are linked by pairs of C—H⋯O hydrogen bonds (Table 1 ▸, Fig. 3 ▸), forming a framework with small cavities of 99 Å^3^, *ca* 5% of the unit-cell volume.

## Database survey   

A search of the Cambridge Structural database (CSD, version 5.39, update May 2018; Groom *et al.*, 2016 ▸) for structures similar to the title compound gave hits including 1*S*,2*S*,4*S*,5*S*)-2,5-bis­[(*p*-toluene­sulfon­yl)amino]­bicyclo­(2.2.1)heptane (Ber­k­essel *et al.*, 2004 ▸), 1,6-anhydro-2,5-dide­oxy-3,4-*O*-iso­propyl­idene-2,5-bis­[(4-methyl­benzene­sulfon­yl)amino]-1-thio­hexitol (Sureshkumar *et al.*, 2005 ▸), (1*R*,3*S*)-1-(toluene­sulfonyl­amido)-3-(toluene­sulfonyl­amido­meth­yl)-3,5,5-tri­methyl­cyclo­hexane (Berkessel *et al.*, 2006 ▸) and *N*,*N*′-propyl­ene­dioxy­bis­(2,4,6-tri-methyl­benzene­sulfonamide) (Wardell *et al.*, 2004 ▸). In the latter compound, the C1—O1 bond length is 1.4448 (19) Å, in agreement with the value found in this study. In addition, the S1—O11 and S1—O12 distances are 1.4312 (12) and 1.4263 (13) Å, respectively and the S1—N1 and S1—C11distances are 1.6608 (14) and 1.7799 (16) Å, respectively.

## Hirshfeld surface analysis   

**Figure 4:**
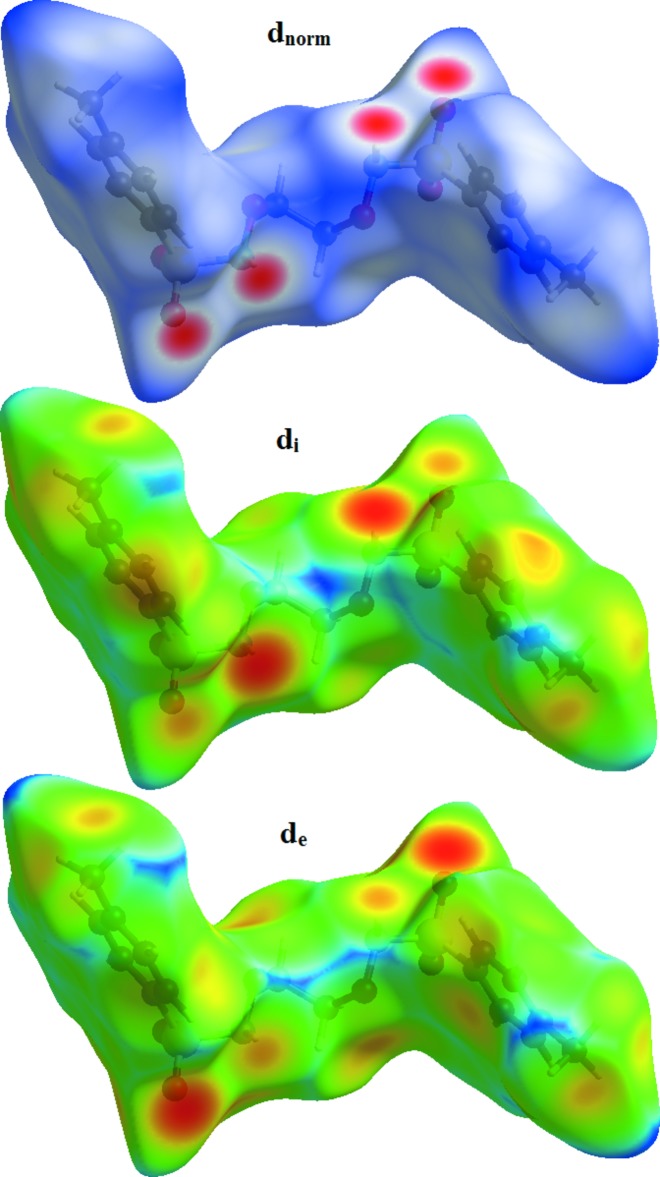
The Hirshfeld surface of the title compound mapped over *d*_norm_, *d*_i_ and *d*_e_.

Hirshfeld surface analysis is a method for visualizing the inter­actions present in the crystal structure and providing qu­anti­tative information about them. The *d*_norm_ representation of the Hirshfeld surface reveals the close contacts of hydrogen-bond donors and acceptors, but other close contacts are also evident. The mol­ecular Hirshfeld surfaces were generated using a standard (high) surface resolution with the three-dimensional *d*_norm_ surfaces mapped over a fixed colour scale of −0.464 (red) to 2.052 (blue) Å using the *CrystalExplorer* (Turner *et al.*, 2017 ▸). The red spots on the surface indicate the inter­molecular contacts involved in the hydrogen bonds. In Figs. 4 ▸ and 5 ▸, the identified red spot is attributed to the H⋯O close contacts which are due to the N—H⋯O hydrogen bonds (Table 1 ▸).

**Figure 6:**
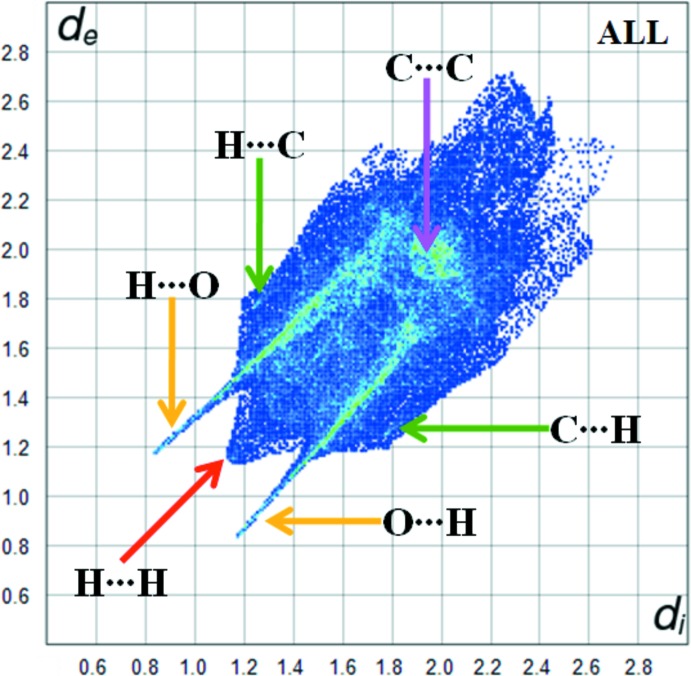
The overall fingerprint plot for the title compound.

**Figure 7:**
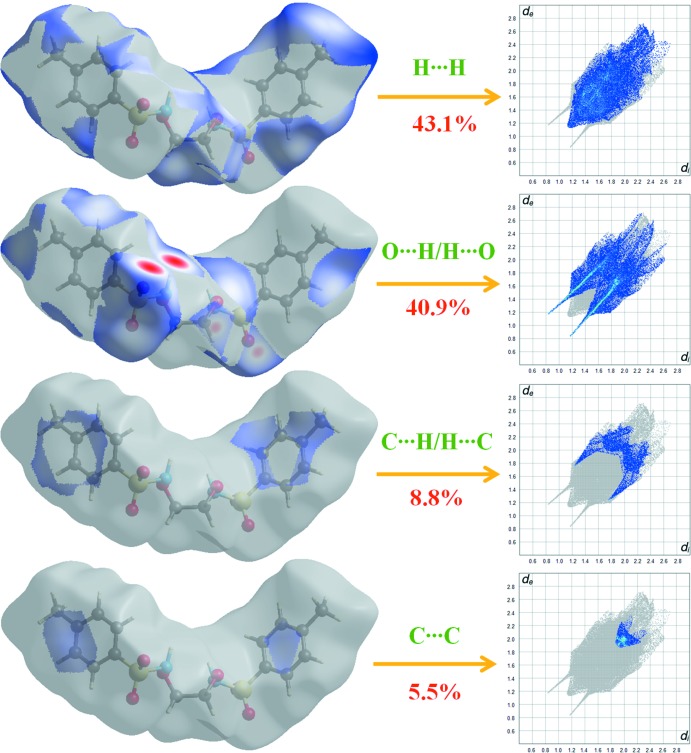
Two-dimensional fingerprint plots with a *d*_norm_ view of the H⋯H (43.1%), O⋯H/H⋯O (40.9%), C⋯H/H⋯C (8.8%) and C⋯C (5.5%) contacts in the title compound.

Fig. 6 ▸ shows the two-dimensional fingerprint of the sum of the contacts contributing to the Hirshfeld surface represented in normal mode. The second plot shown in Fig. 7 ▸ represents the O⋯H/H⋯O contacts (40.9%) between the oxygen atoms inside the surface and the hydrogen atoms outside the surface. *d*_e_ + *d*_i_ ∼2.0 Å and has two symmetrical points at the top, bottom left and right, which is characteristic of an N—H⋯O hydrogen bond.

The H⋯H plot shown in Fig. 7 ▸ shows the two-dimensional fingerprint of the (*d*_i_, *d*_e_) points associated with hydrogen atoms. It is characterized by an end point that points to the origin and corresponds to *d*_i_ = *d*_e_ = 1.08 Å, which indicates the presence of the H⋯H contacts in this study (43.1%). The C⋯H/H⋯C plot in Fig. 7 ▸ shows the contact between the carbon atoms inside the surface and the hydrogen atoms outside the surface of Hirshfeld and *vice versa*. There are two symmetrical wings on the left and right sides (8.8%). Furthermore, there are C⋯C (5.5%), N⋯H/H⋯N (1.4%), O⋯C/C⋯O (0.1%) and S⋯H/H⋯S (0.1%) contacts in the title structure.

**Figure 8:**
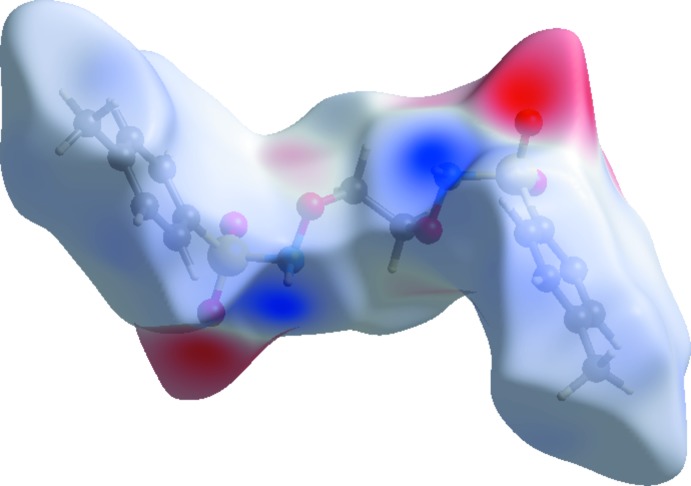
A view of the three-dimensional Hirshfeld surface plotted over electrostatic potential energy.

A view of the three-dimensional Hirshfeld surface of the title compound plotted over electrostatic potential energy in the range −0.095 to 0.123 a.u. using the STO-3G basis set at the Hartree–Fock level of theory is shown in Fig. 8 ▸ where the N—H⋯O hydrogen-bond donors and acceptors are shown as blue and red areas around the atoms related with positive (hydrogen-bond donors) and negative (hydrogen-bond acceptors) electrostatic potentials, respectively.

## Synthesis and crystallization   

The title compound was synthesized according to the method of Bauer & Suresh (1963 ▸). Single crystals (m.p. 414–415 K) were obtained from an ethanol solution (yield 93%)

## Refinement   

Crystal data, data collection and structure refinement details are summarized in Table 2 ▸. C-bound H atoms were positioned geometrically (C—H = 0.93–0.97 Å) and refined as riding, with *U*_iso_(H) = 1.5*U*_eq_(C-methyl) and 1.2*U*_eq_(C) for other H atoms. The NH H atom was located in a difference-Fourier maps and freely refined.

## Supplementary Material

Crystal structure: contains datablock(s) I. DOI: 10.1107/S2056989018017437/xu5951sup1.cif

Structure factors: contains datablock(s) I. DOI: 10.1107/S2056989018017437/xu5951Isup2.hkl

Supporting information file. DOI: 10.1107/S2056989018017437/xu5951Isup3.cml

CCDC reference: 1884045

Additional supporting information:  crystallographic information; 3D view; checkCIF report

## Figures and Tables

**Figure 5:**
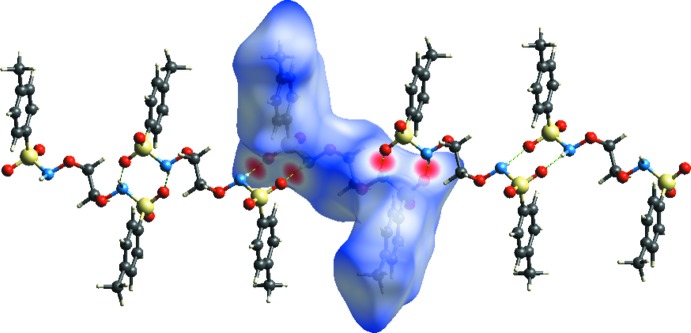
Hirshfeld surface mapped over *d*_norm_ to visualize the inter­molecular inter­actions in the title compound.

**Table 1:** Hydrogen-bond geometry (Å, °)

*D*—H⋯*A*	*D*—H	H⋯*A*	*D*⋯*A*	*D*—H⋯*A*
N1—H1⋯O3^i^	0.83 (3)	2.17 (3)	2.974 (3)	162 (3)
C1—H1*A*⋯O2^ii^	0.97	2.56	3.401 (4)	146

Symmetry codes: (i) 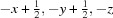; (ii) 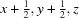.

**Table 2:** Experimental details

Crystal data	
Chemical formula	C_16_H_20_N_2_O_6_S_2_
*M*_r_	400.46
Crystal system, space group	Monoclinic, *C*2/*c*
Temperature (K)	296
*a*, *b*, *c* (Å)	16.3393 (18), 13.4977 (19), 9.7461 (11)
β (°)	113.442 (8)
*V* (Å^3^)	1972.0 (4)
*Z*	4
Radiation type	Mo *K*α
μ (mm^−1^)	0.30
Crystal size (mm)	0.56 × 0.36 × 0.13

Data collection	
Diffractometer	Stoe IPDS 2
Absorption correction	Integration (*X-RED32*; Stoe & Cie, 2002 ▸)
*T*_min_, *T*_max_	0.873, 0.973
No. of measured, independent and observed [*I* > 2σ(*I*)] reflections	6561, 1938, 1070
*R*_int_	0.086
(sin θ/λ)_max_ (Å^−1^)	0.617

Refinement	
*R*[*F* ^2^ > 2σ(*F* ^2^)], *wR*(*F* ^2^), *S*	0.047, 0.116, 0.90
No. of reflections	1938
No. of parameters	123
H-atom treatment	H atoms treated by a mixture of independent and constrained refinement
Δρ_max_, Δρ_min_ (e Å^−3^)	0.14, −0.25

Computer programs: *X-AREA* and *X-RED32* (Stoe & Cie, 2002 ▸), *SHELXL2017* (Sheldrick, 2015 ▸), *ORTEP-3 for Windows* and *WinGX* (Farrugia, 2012 ▸) and *PLATON* (Spek, 2009 ▸).

## supplementary crystallographic information

### Crystal data

**Table:**

C_16_H_20_N_2_O_6_S_2_	*F*(000) = 840
*M**_r_* = 400.46	*D*_x_ = 1.349 Mg m^−^^3^
Monoclinic, *C*2/*c*	Mo *K*α radiation, λ = 0.71073 Å
*a* = 16.3393 (18) Å	Cell parameters from 6407 reflections
*b* = 13.4977 (19) Å	θ = 2.0–27.2°
*c* = 9.7461 (11) Å	µ = 0.30 mm^−^^1^
β = 113.442 (8)°	*T* = 296 K
*V* = 1972.0 (4) Å^3^	Prism, colorless
*Z* = 4	0.56 × 0.36 × 0.13 mm

### Data collection

**Table:**

Stoe IPDS 2 diffractometer	1938 independent reflections
Radiation source: sealed X-ray tube, 12 x 0.4 mm long-fine focus	1070 reflections with *I* > 2σ(*I*)
Detector resolution: 6.67 pixels mm^-1^	*R*_int_ = 0.086
rotation method scans	θ_max_ = 26.0°, θ_min_ = 2.0°
Absorption correction: integration (X-RED32; Stoe & Cie, 2002)	*h* = −20→20
*T*_min_ = 0.873, *T*_max_ = 0.973	*k* = −16→16
6561 measured reflections	*l* = −12→9

### Refinement

**Table:**

Refinement on *F*^2^	0 restraints
Least-squares matrix: full	Hydrogen site location: mixed
*R*[*F*^2^ > 2σ(*F*^2^)] = 0.047	H atoms treated by a mixture of independent and constrained refinement
*wR*(*F*^2^) = 0.116	*w* = 1/[σ^2^(*F*_o_^2^) + (0.0562*P*)^2^] where *P* = (*F*_o_^2^ + 2*F*_c_^2^)/3
*S* = 0.90	(Δ/σ)_max_ < 0.001
1938 reflections	Δρ_max_ = 0.14 e Å^−^^3^
123 parameters	Δρ_min_ = −0.25 e Å^−^^3^

### Special details

**Table:**

Geometry. All esds (except the esd in the dihedral angle between two l.s. planes) are estimated using the full covariance matrix. The cell esds are taken into account individually in the estimation of esds in distances, angles and torsion angles; correlations between esds in cell parameters are only used when they are defined by crystal symmetry. An approximate (isotropic) treatment of cell esds is used for estimating esds involving l.s. planes.

### Fractional atomic coordinates and isotropic or equivalent isotropic displacement parameters (Å^2^)

**Table:**

	*x*	*y*	*z*	*U*_iso_*/*U*_eq_	
S1	0.19812 (4)	0.13241 (6)	0.11223 (9)	0.0759 (3)	
O1	0.04273 (10)	0.15963 (13)	−0.0863 (2)	0.0722 (5)	
C1	0.00029 (17)	0.0748 (2)	−0.1731 (3)	0.0698 (7)	
H1A	−0.060770	0.071765	−0.181199	0.084*	
H1B	0.030688	0.015643	−0.121023	0.084*	
O3	0.28617 (11)	0.15543 (15)	0.1220 (3)	0.0907 (7)	
O2	0.17825 (13)	0.03371 (14)	0.1379 (3)	0.0928 (7)	
N1	0.13462 (14)	0.1594 (2)	−0.0629 (3)	0.0751 (7)	
C2	0.16891 (16)	0.2121 (2)	0.2267 (3)	0.0705 (8)	
C5	0.1182 (2)	0.3366 (3)	0.4043 (4)	0.0929 (10)	
C7	0.1370 (2)	0.1733 (3)	0.3271 (4)	0.1030 (11)	
H7	0.131947	0.105133	0.335264	0.124*	
C4	0.1518 (2)	0.3736 (3)	0.3067 (4)	0.1041 (11)	
H4	0.157573	0.441748	0.299978	0.125*	
C6	0.1129 (3)	0.2367 (4)	0.4145 (5)	0.1153 (13)	
H6	0.092328	0.210422	0.483059	0.138*	
C3	0.1774 (2)	0.3119 (3)	0.2180 (4)	0.0954 (10)	
H3	0.200335	0.338428	0.152627	0.115*	
C8	0.0890 (3)	0.4066 (4)	0.4973 (5)	0.1375 (16)	
H8A	0.083016	0.370639	0.577813	0.206*	
H8B	0.032607	0.435597	0.435712	0.206*	
H8C	0.132692	0.457942	0.537350	0.206*	
H1	0.146 (2)	0.217 (2)	−0.080 (4)	0.089 (11)*	

### Atomic displacement parameters (Å^2^)

**Table:**

	*U*^11^	*U*^22^	*U*^33^	*U*^12^	*U*^13^	*U*^23^
S1	0.0600 (4)	0.0881 (5)	0.0859 (6)	−0.0056 (4)	0.0356 (4)	0.0138 (4)
O1	0.0565 (9)	0.0901 (13)	0.0773 (13)	−0.0033 (9)	0.0344 (9)	−0.0099 (10)
C1	0.0658 (14)	0.0790 (17)	0.0728 (19)	−0.0063 (14)	0.0365 (15)	0.0007 (16)
O3	0.0572 (11)	0.1173 (16)	0.1016 (17)	−0.0056 (10)	0.0358 (11)	0.0226 (13)
O2	0.0875 (13)	0.0809 (13)	0.1204 (19)	−0.0012 (10)	0.0523 (13)	0.0201 (13)
N1	0.0571 (13)	0.0913 (19)	0.0849 (19)	−0.0072 (12)	0.0367 (12)	0.0028 (15)
C2	0.0576 (14)	0.084 (2)	0.0694 (19)	−0.0124 (13)	0.0251 (13)	0.0098 (15)
C5	0.0732 (19)	0.131 (3)	0.065 (2)	−0.008 (2)	0.0175 (16)	−0.010 (2)
C7	0.122 (3)	0.108 (2)	0.100 (3)	−0.038 (2)	0.066 (2)	0.000 (2)
C4	0.124 (3)	0.089 (2)	0.100 (3)	−0.008 (2)	0.044 (2)	0.000 (2)
C6	0.127 (3)	0.148 (4)	0.097 (3)	−0.040 (3)	0.071 (3)	−0.014 (3)
C3	0.107 (2)	0.094 (2)	0.101 (3)	−0.0100 (18)	0.059 (2)	0.011 (2)
C8	0.123 (3)	0.192 (4)	0.093 (3)	0.006 (3)	0.038 (2)	−0.044 (3)

### Geometric parameters (Å, º)

**Table:**

S1—O2	1.4168 (19)	C5—C6	1.357 (5)
S1—O3	1.4376 (17)	C5—C4	1.368 (5)
S1—N1	1.647 (3)	C5—C8	1.512 (5)
S1—C2	1.747 (3)	C7—C6	1.371 (5)
O1—N1	1.426 (2)	C7—H7	0.9300
O1—C1	1.429 (3)	C4—C3	1.379 (5)
C1—C1^i^	1.494 (5)	C4—H4	0.9300
C1—H1A	0.9700	C6—H6	0.9300
C1—H1B	0.9700	C3—H3	0.9300
N1—H1	0.83 (3)	C8—H8A	0.9600
C2—C3	1.361 (4)	C8—H8B	0.9600
C2—C7	1.381 (4)	C8—H8C	0.9600
			
O2—S1—O3	119.02 (12)	C6—C5—C8	122.2 (4)
O2—S1—N1	107.29 (15)	C4—C5—C8	120.0 (4)
O3—S1—N1	102.83 (12)	C6—C7—C2	119.1 (3)
O2—S1—C2	109.02 (13)	C6—C7—H7	120.5
O3—S1—C2	110.20 (13)	C2—C7—H7	120.5
N1—S1—C2	107.80 (13)	C5—C4—C3	121.4 (3)
N1—O1—C1	108.99 (19)	C5—C4—H4	119.3
O1—C1—C1^i^	113.68 (15)	C3—C4—H4	119.3
O1—C1—H1A	108.8	C5—C6—C7	122.2 (3)
C1^i^—C1—H1A	108.8	C5—C6—H6	118.9
O1—C1—H1B	108.8	C7—C6—H6	118.9
C1^i^—C1—H1B	108.8	C2—C3—C4	119.7 (3)
H1A—C1—H1B	107.7	C2—C3—H3	120.2
O1—N1—S1	110.82 (17)	C4—C3—H3	120.2
O1—N1—H1	106 (2)	C5—C8—H8A	109.5
S1—N1—H1	108 (2)	C5—C8—H8B	109.5
C3—C2—C7	119.7 (3)	H8A—C8—H8B	109.5
C3—C2—S1	120.6 (2)	C5—C8—H8C	109.5
C7—C2—S1	119.7 (3)	H8A—C8—H8C	109.5
C6—C5—C4	117.9 (3)	H8B—C8—H8C	109.5
			
N1—O1—C1—C1^i^	−63.7 (3)	C3—C2—C7—C6	1.2 (5)
C1—O1—N1—S1	−108.3 (2)	S1—C2—C7—C6	−178.9 (3)
O2—S1—N1—O1	65.1 (2)	C6—C5—C4—C3	1.8 (6)
O3—S1—N1—O1	−168.64 (18)	C8—C5—C4—C3	−178.7 (3)
C2—S1—N1—O1	−52.2 (2)	C4—C5—C6—C7	−2.4 (6)
O2—S1—C2—C3	−176.8 (3)	C8—C5—C6—C7	178.1 (3)
O3—S1—C2—C3	50.9 (3)	C2—C7—C6—C5	0.9 (6)
N1—S1—C2—C3	−60.6 (3)	C7—C2—C3—C4	−1.7 (5)
O2—S1—C2—C7	3.3 (3)	S1—C2—C3—C4	178.4 (3)
O3—S1—C2—C7	−129.0 (2)	C5—C4—C3—C2	0.2 (6)
N1—S1—C2—C7	119.5 (3)		

Symmetry code: (i) −*x*, *y*, −*z*−1/2.

### Hydrogen-bond geometry (Å, º)

**Table:**

*D*—H···*A*	*D*—H	H···*A*	*D*···*A*	*D*—H···*A*
N1—H1···O3^ii^	0.83 (3)	2.17 (3)	2.974 (3)	162 (3)
C1—H1*A*···O2^iii^	0.97	2.56	3.401 (4)	146

Symmetry codes: (ii) −*x*+1/2, −*y*+1/2, −*z*; (iii) *x*+1/2, *y*+1/2, *z*.
